# Supplementary material for: Associations between Infant Dietary Intakes and Liking for Sweetness and Fattiness Sensations in 8-to-12-Year-Old Children
Source: Nutrients. 2021 Jul 30;13(8):2659. doi: 10.3390/nu13082659 (PMC8399911; doi:10.3390/nu13082659)
Supplement: Supplementary file 1 [file nutrients-13-02659-s001.zip › nutrients-1287938-supplementary.pdf]

Table S1: Percentages of data missingness and methods used for multiple imputation

Variable	Variable type	% of data missingness	Multiple imputation method
**Center**	Binary	0	None
**Children characteristics**			
Sex	Binary	0	None
Age	Continuous	0	None
Gestational age	Continuous	0	None
Birthweight	Continuous	0	None
Age at CF introduction[1]	Continuous	0	None
Any BF duration[1]	Continuous	0	None
Infant dietary intakes			
Carbohydrate	Continuous	0	None
Sugar	Continuous	0	None
Added sugars[1]	Continuous	0	None
Fat	Continuous	0	None
Added fats[1]	Continuous	0	None
Liking in mid-childhood			
Sweetness sensation	Continuous	0	None
Fattiness-and-sweetness sensation	Continuous	0	None
Fattiness sensation	Continuous	0	None
**Maternal characteristics**			
Age at delivery	Continuous	0	None
Primiparous	Binary	<1	Logistic regression
Maternal pre-pregnancy BMI[1]	Continuous	2	Predictive mean matching
Educational attainment (in years)[1]	Continuous	<1	Predictive mean matching
Monthly household income[1]	Continuous	<1	Discriminant function
"Healthy" dietary pattern during pregnancy (pca scores)[2]	Continuous	9	Predictive mean matching
"Western" dietary pattern during pregnancy (pca scores)[2]	Continuous	9	Predictive mean matching

[1]These variables were used as continuous variables in the imputation model and categorized afterwards.
[2]pca: principal component analysis
